# Comparative analysis of *Acomys* *cahirinus* and *Mus* *musculus* responses to genotoxicity, oxidative stress, and inflammation

**DOI:** 10.1038/s41598-023-31143-4

**Published:** 2023-03-09

**Authors:** Lamees N. Ghebryal, Magda M. Noshy, Akmal A. El-Ghor, Shaymaa M. Eissa

**Affiliations:** Department of Zoology, Faculty of Science, Cairo University, Giza, 12613 Egypt

**Keywords:** Biochemistry, Genetics, Molecular biology, Zoology

## Abstract

The Egyptian spiny mouse, *Acomys* *cahirinus*, is a recently described model organism for regeneration studies. It has surprising powers of regeneration with relatively fast repairing mechanisms and reduced inflammation form compared to other mammals. Although several studies have documented the exceptional capabilities of *Acomys* to regenerate different tissues after injury, its response to different cellular and genetic stresses is not yet investigated. Therefore, the current study aimed to investigate *Acomys* abilities to resist genotoxicity, oxidative stress and inflammation induced by acute and subacute treatments with lead acetate. Responses of *Acomys* were compared with those of the lab mouse (*Mus* *musculus*), which displays signatures of the “typical” mammalian response to various stressors. Cellular and genetic stresses were induced by using acute and subacute doses of Lead acetate (400 mg/kg and 50 mg/kg for 5 days, respectively). The assessment of genotoxicity was carried out by using comet assay, while oxidative stress was evaluated by measuring the biomarkers; MDA, GSH and antioxidant enzymes CAT and SOD. Moreover, inflammation was assessed by analyzing the expression of some inflammatory-regeneration-related genes: *CXCL1*, *IL1-β*, and *Notch* *2* and immunohistochemical staining of TNF-α protein in brain tissue, in addition to histopathological examination of brain, liver, and kidneys. The obtained results revealed a unique resistance potency of *Acomys* to genotoxicity, oxidative stress, and inflammation in certain tissues in comparison to *Mus*. Altogether, the results revealed an adaptive and protective response to cellular and genetic stresses in *Acomys*.

## Introduction

The molecular or cellular events taken in response to damage tend to differ between organisms. Some tend to be more resistant to biological stress than others, thus would have higher potentials to repair the damage, eliminate the stress, and ensure the organism’s survival. These potentials were known to be relatively limited in mammals when discussing the healing mechanisms. However, one remarkable mammal has been recently discovered with some exceptional capabilities of healing and regeneration: the Egyptian spiny mouse (*Acomys* *cahirinus*)^1^. Its regeneration capabilities were first documented experimentally by Seifert et al.^2^. When compared with the rest of the mammals, *A.* *cahirinus* showed more improved regeneration responses. It was able of fully recovery after harmful damage, showed an epimorphic scar-free regeneration of damaged tissues, retrieved organs’ functionality in relatively brief periods of time, and expressed a blunted immune system with reduced inflammatory reactions^1–4^.

With those outstanding features, *Acomys* species has become the most powerful tool for regenerative medicine, healing, and stress-resistance studies, and turned into subject of interest for broad range of research fields. Multiple injury-test experiments were performed on different tissues of *A.* *cahirinus* *such* *as* skin^5–11^, ear^12,13^, *Mus*cles^14,15^, heart^16–18^, spinal cord^19^, kidneys^20^. However, it has not been investigated yet whether these abilities were also applied on the cellular and genetic stresses or not. Thus, in the present study, we are investigating how *A.* *cahirinus* would respond to cellular and genotoxic stimuli.

Harmful agents as toxicants tend to cause different forms of damage to organisms, eventually causing biological stress to different biological levels of the body, accompanied with a form of imbalance to the homeostatic state of it. One of such effects can be seen at the molecular level as direct damage to DNA causing genotoxicity^21^. The destruction of the genetic material of the cell can cause profound changes to gene product, causing numerous pathologies that might be eventually lethal, thus the study of DNA damage responses is considered important for disease management^22,23^. Moreover, indirect genotoxicity can also result from toxicants by causing oxidative stress^21^. Oxidative stress is known as an imbalance between the free reactive oxygen species (ROS) produced in the body and the biological system’s ability to detoxify and repair this damage with antioxidants^24^. ROS are normally produced by the body to play several physiological roles, however, when their levels are elevated, cells, tissues, and even the genetic material can get damaged. In order to prevent such consequences, the body has an antioxidant defensive system to protect it from ROS-induced damage. On the other hand, toxicants and several other environmental stressors can greatly contribute to the overproduction of the ROS, when this happens, genotoxic and oxidative stress results.

The variability in the organisms’ ability to resist genotoxicity including oxidative stress confirms the variation in genetic regulation of stress^25^. For this reason, we aimed to investigate if *Acomys* could have potential to resist genotoxicity, oxidative stress and inflammation induced by treatment with lead acetate. These potentials were compared with those of ordinary lab mouse *Mus* *musculus.* We used Lead acetate (LA) as an inducer of genotoxicity, oxidative stress, and inflammation^26–28^. LA effect was assessed in three of its target organs: brain, liver, and kidneys. Parameters evaluated were the Comet assay for genotoxic estimation at DNA level, the MDA, reduced GSH and antioxidant enzymes catalase (CAT) and sodium oxide dismutase (SOD) parameters for oxidative stress, and the expression of some inflammatory-regeneration-related genes: *CXCL1*, IL1-*β*, and *Notch* *2* and immunohistochemical staining of TNF-α protein in brain tissue. The three tissues were also assessed with histopathological examination.

## Results

### Responses of *Acomys *and *Mus* to acute treatment (single dose; 400 mg/kg) of LA

#### Comet assay

Table 1 showed that treatment with LA single dose induced significant (*P* < 0.05) increases in all studied comet parameters of *Acomys* and *Mus* as compared to their respective controls. The % change values of TM showed that the extent of DNA damage was lower in *Acomys* brain and liver (89.7%and 230%, respectively) compared to *Mus* (161.4% and 261%, respectively).

**Table 1:** Comet assay parameters analyzed in the brain, liver, and kidneys of *Acomys* *cahirinus* and *Mus* *musculus* following single dose (400 mg/kg) of LA.

Parameter	Tissue	Species	Experimental groups		
			Control	Treated	% change
TL (μm)	Brain	*Acomys* *cahirinus*	2.66 ± 0.18	4.09 ± 0.34*	53.76
		*Mus* *musculus*	3.15 ± 0.16	5.14 ± 0.37*	63.17
	Liver	*Acomys* *cahirinus*	2.80 ± 0.17	4.61 ± 0.29*	64.64
		*Mus* *musculus*	3.36 ± 0.29	6.94 ± 0.47*	106.55
	Kidney	*Acomys* *cahirinus*	2.59 ± 0.10	5.15 ± 0.64*	98.84
		*Mus* *musculus*	3.86 ± 0.40	6.87 ± 0.38*	77.98
Tail DNA%	Brain	*Acomys* *cahirinus*	14.60 ± 0.62	18.05 ± 0.99*	23.63
		*Mus* *musculus*	14.08 ± 0.15	22.42 ± 0.38*	59.23
	Liver	*Acomys* *cahirinus*	11.63 ± 0.49	23.60 ± 0.43*	102.92
		*Mus* *musculus*	21.33 ± 1.50	28.65 ± 0.92*	34.32
	Kidney	*Acomys* *cahirinus*	12.61 ± 0.51	21.75 ± 0.57*	72.48
		*Mus* *musculus*	18.78 ± 0.61	22.64 ± 0.95*	20.55
TM	Brain	*Acomys* *cahirinus*	0.39 ± 0.11	0.74 ± 0.09*	89.70
		*Mus* *musculus*	0.44 ± 0.08	1.15 ± 0.04*	161.40
	Liver	*Acomys* *cahirinus*	0.33 ± 0.02	1.09 ± 0.17*	230.00
		*Mus* *musculus*	0.55 ± 0.18	1.99 ± 0.25*	261.80
	Kidney	*Acomys* *cahirinus*	0.33 ± 0.06	1.20 ± 0.18*	264.00
		*Mus* *musculus*	0.72 ± 0.18	1.56 ± 0.04*	117.00

Data displayed as mean ± standard error of mean. (*) represent significant differences as compared to the corresponding controls (*P* < 0.05).

*TL* tail length, *TM* tail moment.

#### Oxidative stress

**Figure 1:**
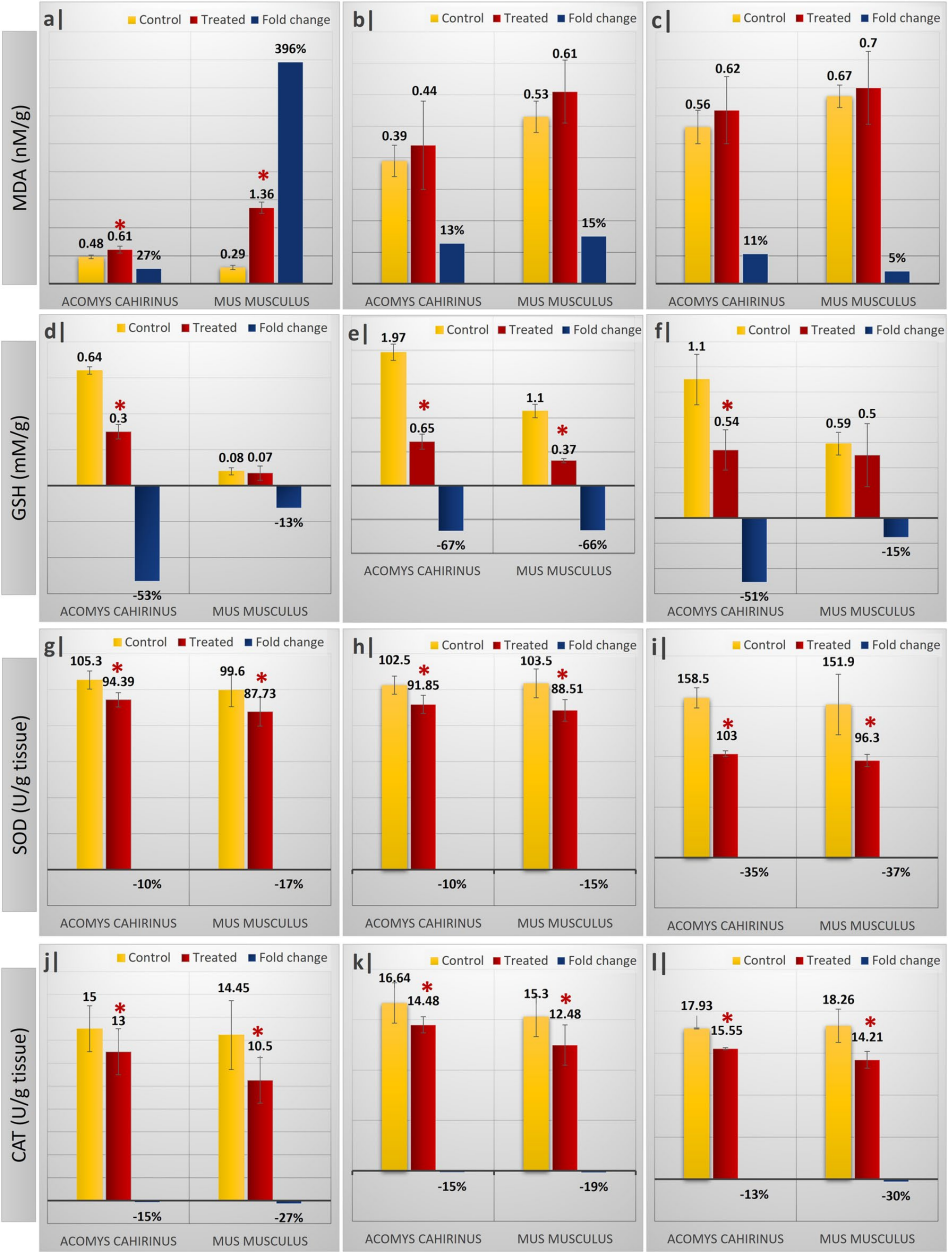
Effect of acute administration of LA (single dose; 400 mg/kg) on oxidative stress biomarkers: MDA brain (**a**)–liver (**b**)–kidneys (**c**), GSH content of brain (**d**)–liver (**e**)–kidneys (**f**), SOD activity of brain (**g**)–liver (**h**)–kidney (**i**) and finally CAT activity of brain (**j**)–liver (**k**)–kidney (**l**) of control and treated experimental groups of *Acomys* *cahirinus* and *Mus* *musculus*. Data presented as mean ± standard error of mean. Fold change represents the percentage change relative to the corresponding control groups. Asterisk: significant (*p* < 0.05) difference as compared with the corresponding control groups.

All LA-treated groups of *Acomys* and *Mus* showed elevated levels of MDA compared to their respective controls (Fig. 1a–c). However, this elevation was significant (*p* < 0.05) only in the brain tissue (Fig. 1a). The induction of MDA after LA treatment in brain tissue of *Acomys* is lower than *Mus* (% change 27% and 396%, respectively). On the other hand, the brain, liver, and kidneys contents of GSH were always remarkably lower in all LA-treated groups for both genera compared to their respective controls (Fig. 1d–f). The data of percent change (Fig. 1d–f) revealed that the depletion of GSH levels in the treated groups was pronounced in the *Acomys* compared to the *Mus*. In addition, the % change of both antioxidant enzymes SOD and CAT (Fig. 1g–l) was higher in *Mus* compared to *Acomys* in the three studied tissues.

#### Gene expression analysis

**Figure 2:**
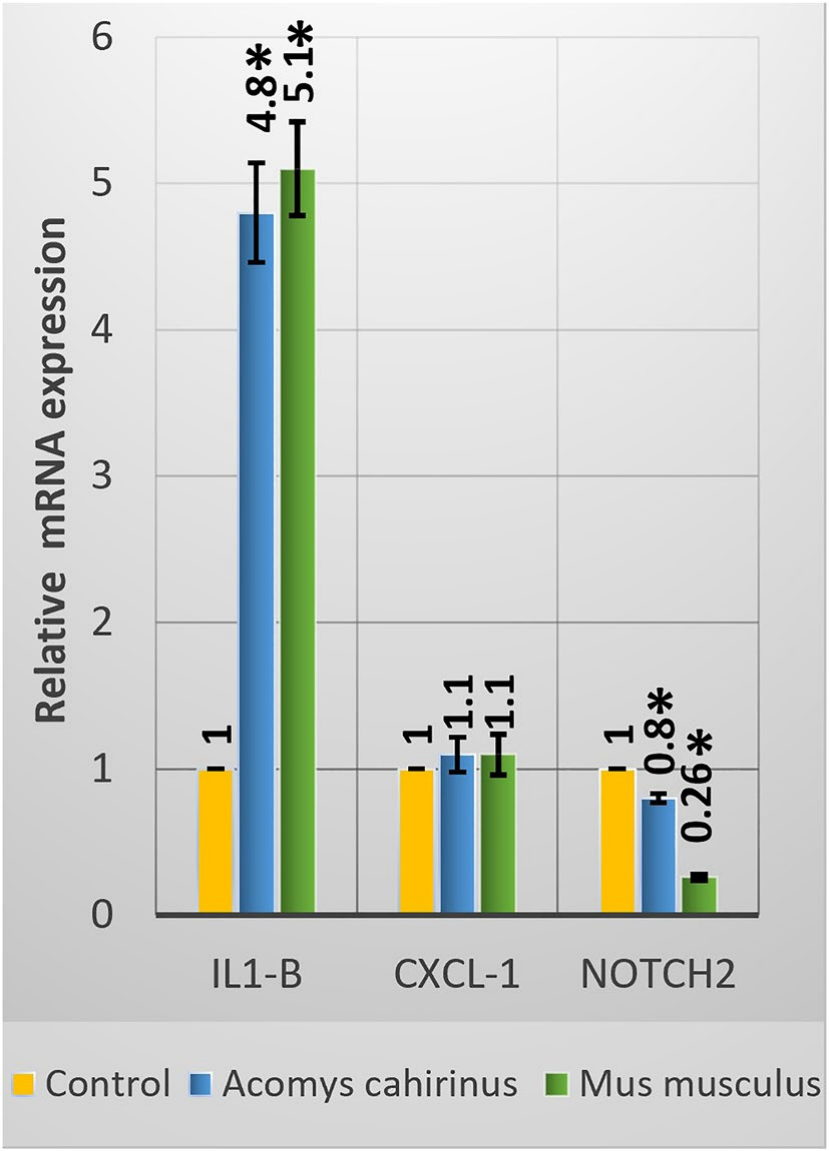
Gene expression analysis of *IL1-β*, *CXCL-1*, and *Notch* *2* in brain samples of *Acomys* *cahirinus* and *Mus* *musculus*, following acute administration of LA. Data presented as mean ± standard error of mean. Asterisk: significant (*p* < 0.05) difference as compared with the corresponding control groups.

Figure 2 revealed that treatment with the acute dose of LA caused significant upregulation of *IL1-B* in both brain tissues of *Acomys* and *Mus*; however, the response for the upregulation was higher in *Mus*. Although the expression of the *CXCL-1* was also upregulated in *Acomys* and *Mus,* both increases were nonsignificant. In contrast, the expression of *Notch* *2* was significantly downregulated in *Acomys* and *Mus,* but the downregulation was higher in *Mus* (74%) than *Acomys* (20%) compared to their corresponding controls.

#### Histopathological examination

**Figure 3:**
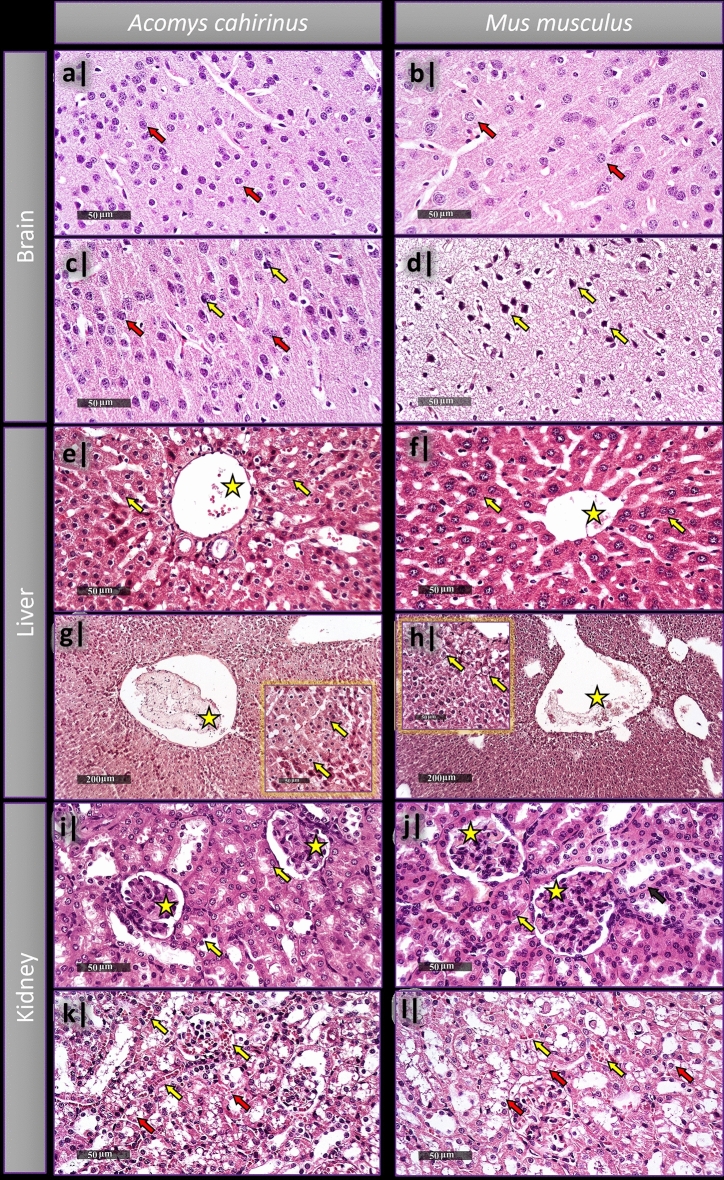
Histopathological examination of brain (**a–d**), liver (**e–h**), and kidneys (**i–l**) of *Acomys* *cahirinus* and *Mus* *musculus* following single acute administration of LA. (**a,b**) Cortical region of brain in normal control samples of *Acomys* (**a**) and *Mus* (**b**) shows intact nuclear details (red arrows). (**c**) Treated *Acomys* group sample shows almost intact cortical neurons (red arrows) with intact intercellular brain matrix, and few abnormal degenerative changes (yellow arrows). (**d**) Treated *Mus* group sample with severe neuronal degenerative changes observed as structureless angular cell bodies (Yellow arrows) and severe vacuolization of brain matrix. (**e,f**) normal control liver samples of *Acomys* (**e**) and *Mus* (**f**), both demonstrate the common intact features of hepatic tissue with intact subcellular details (yellow arrows) and hepatic vasculatures (yellow star). (**g**) Treated *Acomys* group sample shows severe dilatation of hepatic central veins (yellow star), moderate degenerative changes with multiple pycnotic nuclei (yellow arrows). (**h**) Treated *Mus* group sample also shows severe dilatation of central hepatic vein (yellow star) with diffuse vacuolar degenerative changes (yellow arrows) all over different zones of hepatic lobules. (**i,j**) Normal control kidneys samples of *Acomys* (**i**) and *Mus* (**j**) show the common histological features of renal parenchyma with intact renal corpuscles (yellow stars) and tubular epithelium (yellow arrows). Both treated groups of *Acomys* (**k**) and *Mus* (**l**) show severe necrotic and degenerative changes in the renal tubular epithelium with lost luminal border integrity (red arrows) and mild congested intratubular blood vessels.

Microscopic examination of different layers of cortical region of *Acomys* and *Mus* control groups showed well organized apparent intact neurons, intact subcellular and nuclear details, as well as intact intercellular brain matrix with minimal glial cell infiltrates (Fig. 3a,b). Treated *Acomys* groups showed only minimal records of abnormal degenerative changes, the rest was mostly intact (Fig. 3c), unlike the *Mus* treated groups whose samples showed severe neuronal degenerative changes allover cortical layers accompanied with severe vacuolization of brain matrix (Fig. 3d). Normal control liver samples of both strains demonstrated normal intact histological features of liver parenchyma, hepatocytes, hepatic vasculatures, and hepatic sinusoids (Fig. 3e,f). Both treated *Acomys* and *Mus* groups showed severe dilatation of hepatic central veins with minimal inflammatory cells infiltrates (Fig. 3g,h). *Acomys* showed moderate degenerative and necrotic changes in perivascular hepatocytes (Fig. 3g) while *Mus* showed vacuolar degenerative changes widespread all-over different zones of the hepatic lobules (Fig. 3h). Kidneys samples of all the control groups have also demonstrated normal intact histological features of renal parenchyma, renal corpuscles, renal tubules with almost intact tubular epithelium (Fig. 3i,j), on the other hand, both species’ samples showed severe degenerative and necrotic changes of renal tubular epithelium, mild congested glomerular tufts and intratubular blood vessels (Fig. 3k,l).

### Responses of *Acomys *and *Mus* to subacute treatment (multiple doses; 50 mg/kg) of LA

#### Comet assay

The data of comet assay (Table 2) showed significant (*P* < 0.05) increases in TL and TM of LA-treated groups of both *Acomys* and *Mus* compared to their corresponding controls. The values of the percent change (% of change relative to corresponding control) of TL, tail DNA%, and TM showed that the extent of DNA damage was lower in *Acomys* brain and kidneys compared to *Mus* (Table 2) and the attached supplementary figures (1, 2 & 3) illustrate the extent of DNA damage in the three examined tissues (supplementary file).

**Table 2:** Comet assay parameters analyzed in the brain, liver, and kidneys of *Acomys* *cahirinus* and *Mus* *musculus* following multiple doses (50 mg/kg for five consecutive days) of LA.

Parameter	Tissue	Species	Experimental groups		
			Control	Treated	% change
TL (μm)	Brain	*Acomys* *cahirinus*	2.61 ± 0.12	4.02 ± 0.37*	54.00
		*Mus* *musculus*	3.12 ± 0.01	5.59 ± 0.07*	79.20
	Liver	*Acomys* *cahirinus*	2.16 ± 0.04	3.57 ± 0.27*	65.30
		*Mus* *musculus*	3.40 ± 0.35	4.84 ± 0.26*	42.40
	Kidney	*Acomys* *cahirinus*	2.06 ± 0.05	3.79 ± 0.31*	83.98
		*Mus* *musculus*	4.26 ± 0.61	8.88 ± 0.98*	109.00
Tail DNA%	Brain	*Acomys* *cahirinus*	14.96 ± 0.90	17.55 ± 0.34	17.30
		*Mus* *musculus*	14.33 ± 0.67	18.41 ± 1.29*	28.50
	Liver	*Acomys* *cahirinus*	11.02 ± 0.40	19.93 ± 1.03*	80.85
		*Mus* *musculus*	18.68 ± 0.27	20.10 ± 3.27	7.60
	Kidney	*Acomys* *cahirinus*	11.44 ± 0.79	14.38 ± 2.78	25.70
		*Mus* *musculus*	12.42 ± 1.37	29.42 ± 2.50*	137.00
TM	Brain	*Acomys* *cahirinus*	0.39 ± 0.08	0.71 ± 0.10*	82.10
		*Mus* *musculus*	0.45 ± 0.07	1.03 ± 0.01*	129.00
	Liver	*Acomys* *cahirinus*	0.24 ± 0.02	0.71 ± 0.15*	196.00
		*Mus* *musculus*	0.64 ± 0.14	0.97 ± 0.07*	52.00
	Kidney	*Acomys* *cahirinus*	0.24 ± 0.03	0.54 ± 0.05*	125.00
		*Mus* *musculus*	0.53 ± 0.04	2.61 ± 0.73*	392.00

Data displayed as mean ± standard error of mean. (*) represent significant (P < 0.05) differences as compared to the corresponding controls.

*TL* tail length, *TM* tail moment.

#### Oxidative stress

**Figure 4:**
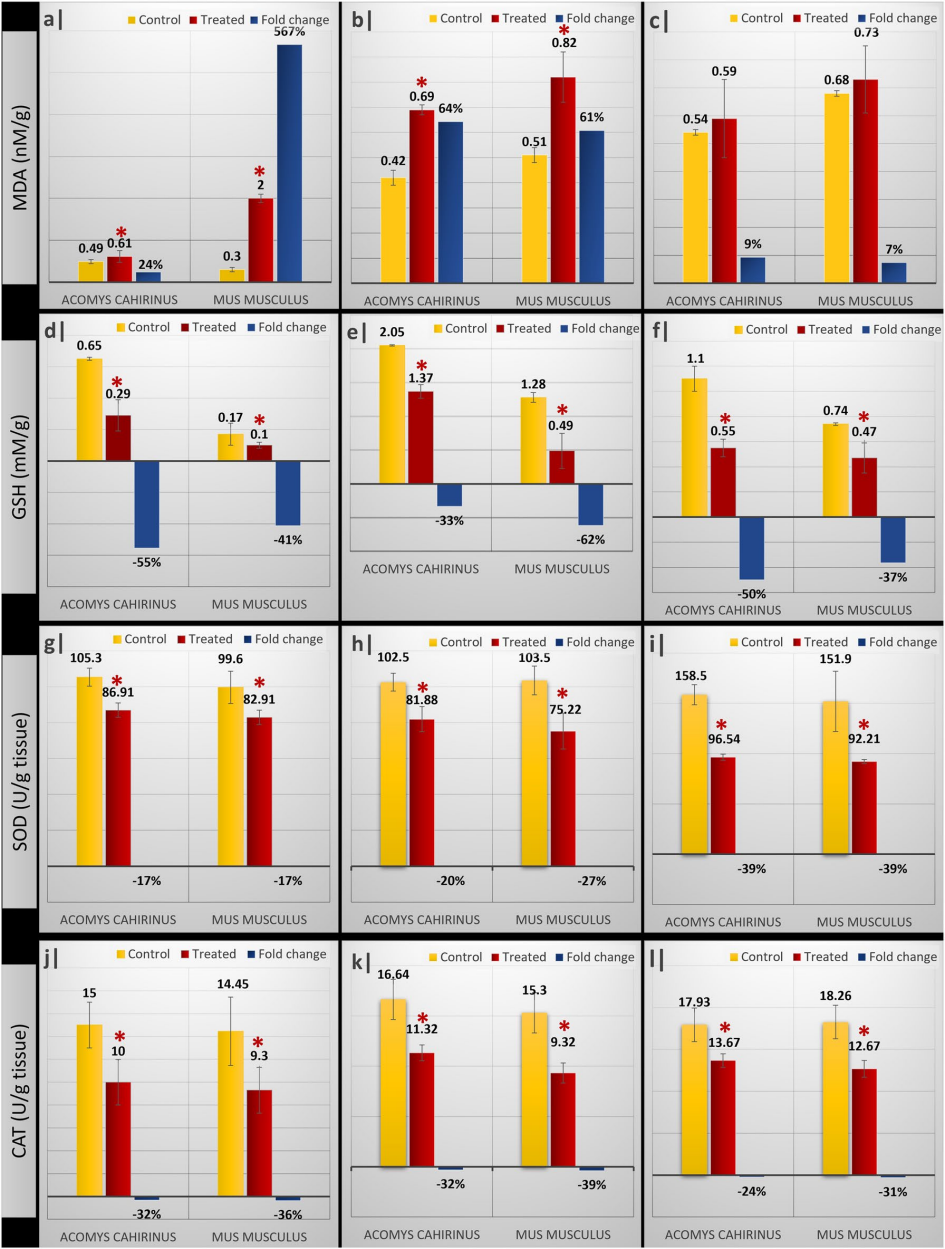
Effect of subacute administration of LA (multiple doses; 50 mg/kg for five consecutive days) on oxidative stress biomarkers: MDA brain (**a**)–liver (**b**)–kidneys (**c**), GSH content of brain (**d**)–liver (**e**)–kidneys (**f**), SOD activity of brain (**g**)–liver (**h**)–kidney (**i**) and finally CAT activity of brain (**j**)–liver (**k**)–kidney (**l**) of control and treated experimental groups of *Acomys* *cahirinus* and *Mus* *musculus*. Data presented as mean ± standard error of mean. Fold change represents the percentage change relative to the corresponding control groups. Asterisk: significant (*p* < 0.05) difference as compared with the corresponding control groups.

Compared to the corresponding controls, MDA levels were elevated in all studied tissues of the LA-treated groups of both genera, but these elevations were only significant in the brain and liver tissues. The MDA content in brain tissue of *Acomys* was extremely lower compared to *Mus* reflecting high potential to resist oxidative stress. On the other hand, GSH contents of all studied tissues of both genera were significantly reduced compared to their corresponding controls (Fig. 4). Compared to the corresponding controls, the antioxidant enzymes SOD and CAT activity was significantly decreased in all studied tissues in both animal species, while the % change was higher in *Mus* than in *Acomys* (Fig. 4g–l).

#### Gene expression analysis (B)

**Figure 5:**
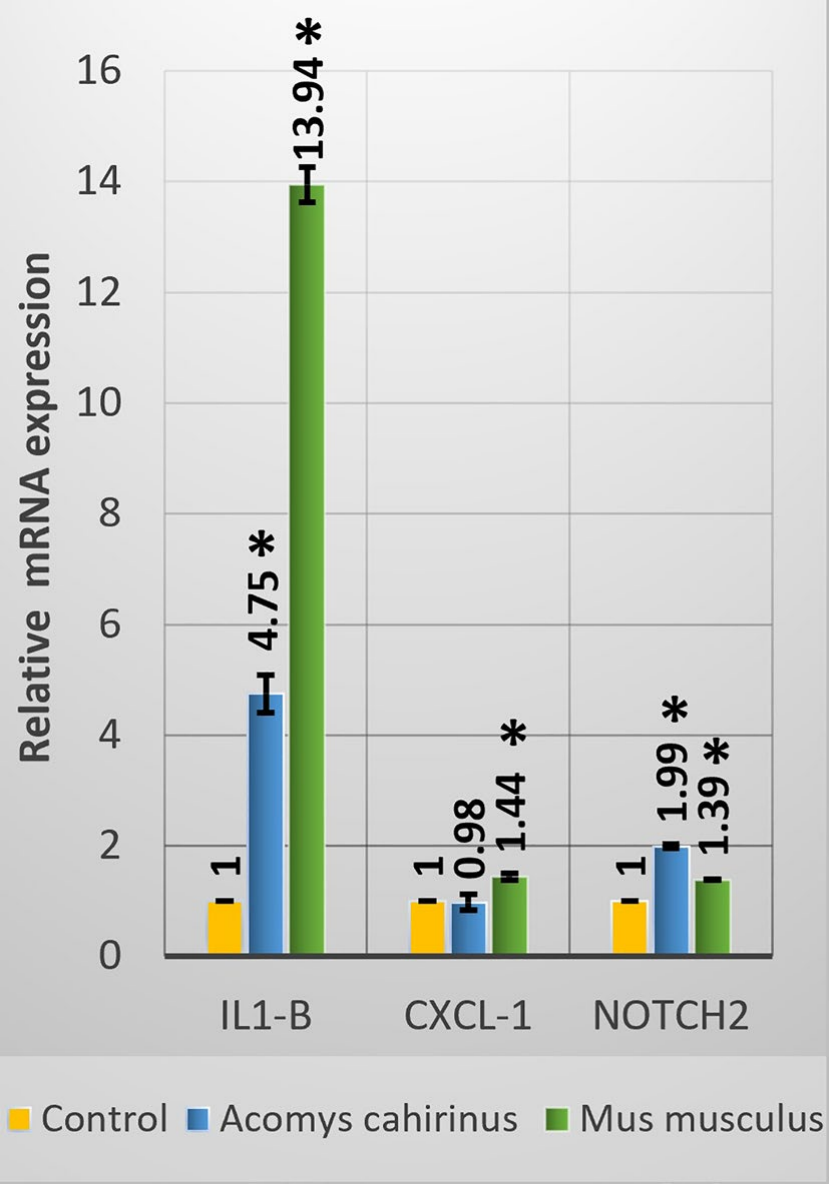
Gene expression analysis of *IL1-β*, *CXCL-1*, and *Notch* *2* in brain samples of *Acomys* *cahirinus* and *Mus* *musculus* following subacute administration of LA relative to the corresponding controls standardized to one. Data presented as mean ± standard error of mean. Asterisk: significant difference where *p* < 0.05.

Compared with the corresponding controls, the expression of *IL1-β* in the brain samples of *Acomys* and *Mus* treated groups were significantly (*p* < 0.05) upregulated by 375% and 1294% respectively. The mRNA expression level of *CXCL-1* in *Mus* after exposure to Pb acetate was significantly) higher compared to the control. On contrary, the gene expression level of *CXCL-1* in *Acomys* administered LA was insignificantly lower than in the controls. In *Acomys* and *Mus* exposed to 50 mg/kg LA, the gene expression level of *Notch* *2* was elevated by 99% and 39% respectively as compared to their respective controls (Fig. 5).

#### Histopathological examination (B)

**Figure 6:**
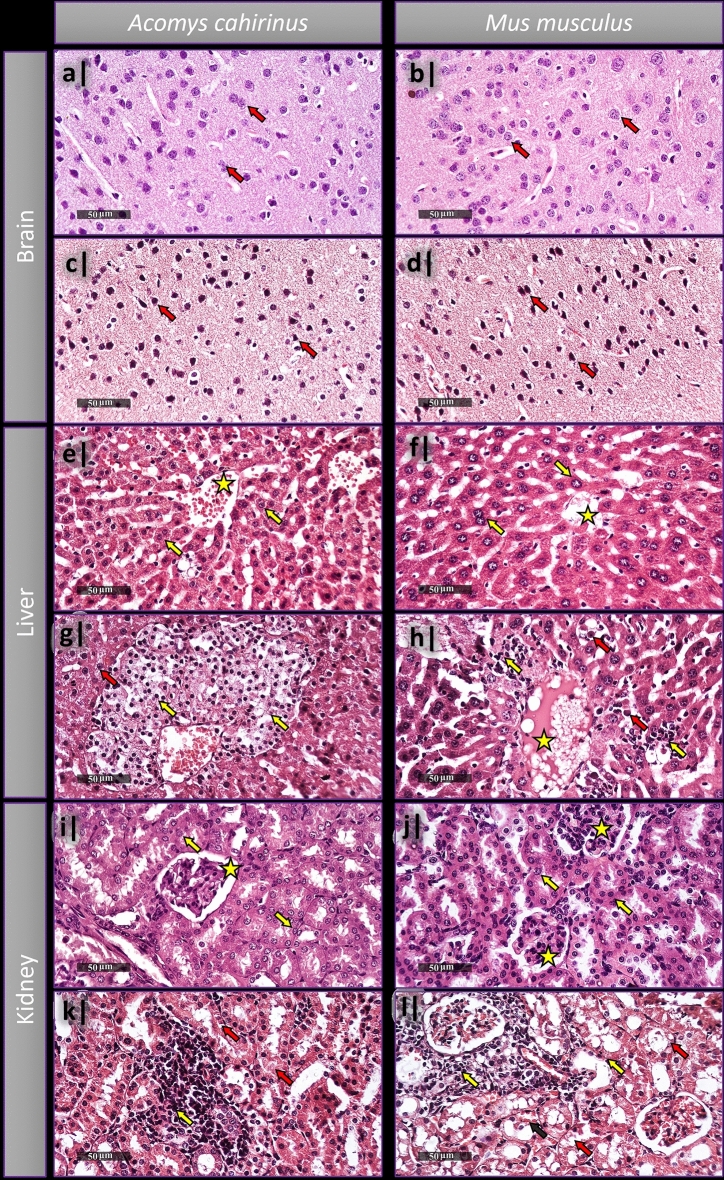
Histopathological examination of brain (**a–d**), liver (**e–h**), and kidneys (**i–l**) of *Acomys* *cahirinus* and *Mus* *musculus* treated with 50 mg/kg LA for 5 consecutive days. (**a,b**) Brain cortical region of *Acomys* (**a**) and *Mus* (**b**) shows intact nuclear details (red arrows). Both treated groups of *Acomys* (**c**) and *Mus* (**d**) show severe neuronal degenerative changes all over the cortical layers accompanied with multiple angular pyknotic structureless cell bodies (red arrows) in addition to severe vacuolization of brain matrix. (**e,f**) Normal control liver samples of *Acomys* (**e**) and *Mus* (**f**), both show intact subcellular details of hepatic tissues (yellow arrows) and hepatic vasculatures (yellow star). (**g**) Treated *Acomys* group sample shows severe vacuolar degenerative changes of hepatocytes (red arrow) with pyknotic nuclei (yellow arrows) all over hepatic lobules. (**h**) Treated *Mus* group sample shows severe dilatation hepatocytes (yellow star) with perivascular inflammatory cell filtrates (yellow arrows) and apoptotic bodies formation (red arrows). (**i,j**) Normal control kidneys samples of *Acomys* (**i**) and *Mus* (**j**) show the intact renal corpuscles (yellow stars) and tubular epithelium (yellow arrows). (**k**) Treated *Acomys* kidney samples show mild records of degenerated tubular epithelium (red arrows) with occasional periglomerular inflammatory cells infiltrates (yellow arrow) compared with the more severe records of the degenerated tubular epithelium in the *Mus* (**l**) (red arrow), more congested blood vessels (black arrow), and occasional periglomerular inflammatory cell infiltrates (yellow arrows).

Brain cortical region of *Acomys* and *Mus* (Fig. 6a,b) respectively, show intact nuclear details. The effect of the 50 mg/kg subacute doses of LA had almost similar effects as the 400 mg/kg single acute dose in brain cortex of the experimental groups (Fig. 6c,d). Normal control liver samples of *Acomys* and *Mus* (Fig. 6e,f) respectively, both show intact subcellular details of hepatic tissues and hepatic vasculatures. On the other hand, the effect on the liver of the treated group of *Mus* was observed as severe dilatation in the hepatic vasculatures, moderate activated Kupffer cells in hepatic sinusoids accompanied with many figures of apoptotic bodies formation and perivascular inflammatory cells infiltrates (Fig. 6h). While in the *Acomys* there were more severe degenerative changes in hepatocytes with pyknotic nuclei allover hepatic lobules (Fig. 6g). The histological examination of the kidney’s samples of normal control *Acomys* and *Mus* show the intact renal corpuscles and tubular epithelium (Fig. 6i,j). While the subacute treatment showed more severe records of degenerative tubular epithelium changes, as well as higher congested BVs in the *Mus* than the *Acomys*. Occasional periglomerular inflammatory cells infiltrates were observed too in both strains (Fig. 6k,l).

#### Immunohistochemical examination

**Figure 7:**
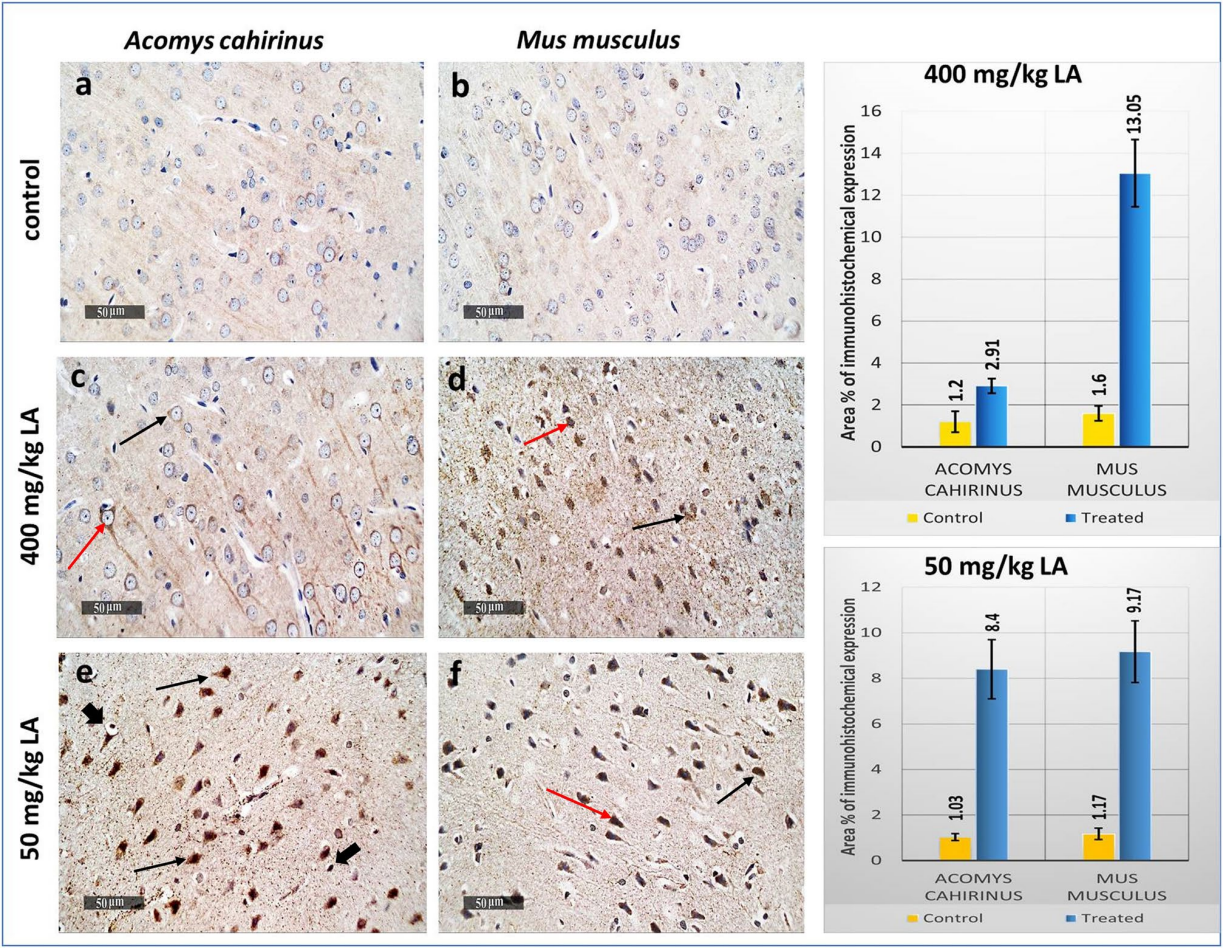
Photomicrographs represent immunohistochemistry staining of TNF alpha is evident in outer granular neurons (black arrow) and outer pyramidal neurons (red arrow) with co-existence in associated glial cells (arrowhead). (**a**) Normal control brain cortex of *Acomys*. (**b**) Normal control brain cortex of *Mus*. (**c,d**) Brain cortex sections of (400 mg/kg LA) treated *Acomys* and *Mus* respectively. (**e,f**) Brain cortex sections of *Acomys* and *Mus* treated with (50 mg/kg LA) for five consecutive days.

Compared with the corresponding controls, the protein expression of TNF-α in the cortical region of brain samples of *Acomys* and *Mus* treated groups were significantly (*p* < 0.05) increased. The expression of TNF-α in the cortex of *Mus* of both treatment (acute and subacute) was significantly upregulated compared to the treated groups of *Acomys* Fig. 7.

## Discussion

The spiny mouse, *Acomys* *cahirinus* is a recently described model organism for regeneration studies. It displays surprising powers of regeneration among mammals because it does not form a scar (fibrose) in response to tissue injury as most other mammals do^4^. Although several studies have documented the regeneration capabilities of *Acomys* after different tissue injuries, the response of *Acomys* to different cellular and genetic stresses is not yet investigated. This study aimed to investigate if *Acomys* could have potential to resist genotoxicity, oxidative stress and inflammation induced by treatment with lead acetate. These potentials were compared with those of ordinary lab mouse *Mus* *musculus*, which has a typical mammalian response to various stressors. Several studies reported that LA is a stressor that can elicit cellular stress response in mice. These stresses include genotoxic stress^28–30^, oxidative stress^31–33^, and inflammatory responses^34,35^. Accordingly, in the present study LA was chosen for its documented ability to induce genotoxicity, oxidative stress, and inflammation.

The in vivo single cell gel electrophoresis, also called comet assay used in the present study was a very sensitive method to detect single breaks of DNA and determination of genotoxic potential of toxic agents^36^. Treatment with LA either acute or subacute doses induced DNA damage in both genera (*Acomys* and *Mus*) as indicated by the significant increase (*p* < 0.05) in tail moment (TM) values of the comet assay as compared to their respective controls. Mohamed et al*.* (2017) attributed genotoxicity to variable toxins that interact with the DNA structure and its sequence causing either direct damage by altering the DNA repair mechanism, or indirect damage due to gene and protein alternation or formation of reactive oxygen species (ROS)^21^. ROS were believed to cause genotoxicity not only indirectly by disrupting the homeostasis between the oxidants and antioxidants, but also directly by interacting with the nucleophilic centers within the cell, eventually this damage would be observed as single or double DNA strand breaks or DNA adduct formation^37^. In this study, LA was used for its direct and indirect damaging effects^38^.

The results of comet assay (% change values of tail moment (TM) revealed that acute LA treatment induced lower DNA damage in *Acomys* compared to *Mus* in brain and liver tissues. On the contrary, acute LA treatment induced higher DNA damage in *Acomys* kidneys compared to *Mus*. This suggests that *Acomys* kidneys are more sensitive to acute LA treatment. Dickinson et al*.* (2007) reported that spiny mice have a significantly reduced kidney mass and volume, and fewer glomeruli, compared with laboratory mice^39^. This is likely to affect the filtration surface area and capacity for glomerular ultrafiltration of the kidney. These structural and functional differences may account for the increased DNA damage observed in kidney tissue of *Acomys*. Moreover, kidneys play role in mediating toxicity of multiple drugs, and that lead is one of the nephrotoxicants^40^. *Acomys* kidneys possibly could not accommodate the large dose of LA in 24 h. For the groups treated with subacute doses of LA, the results showed that the potential capacity of *Acomys* to resist DNA damage is higher than *Mus* in brain and kidneys but not in liver. The higher DNA damage observed in *Acomys* liver cannot be explained.

Although treatment with acute and subacute doses of LA created genotoxic stress in both genera, there was a tangible difference of *Acomys* being normally more immune within its natural habitats observed as lower DNA damage in brain, liver, and kidney samples of the untreated control groups compared to those of *Mus*. GSH natural contents in brain, liver, and kidneys of untreated controls were also found to be higher in *Acomys* compared to *Mus*, this could indicate stronger defense system against oxidants in *Acomys* more than *Mus*, this goes in line with observations and explanations of previous studies^41–44^.

Oxidative stress was defined as an imbalance between oxidants and antioxidants in favor of oxidants. In the present study, oxidative stress biomarkers were used: determination of malondialdehyde (MDA), reduced glutathione (GSH) and antioxidant enzymes CAT and SOD. MDA is one of the most commonly studied end products for lipid peroxidation that is used for estimation of oxidative stress^45^. Moreover, GSH is an important peptide in redox management and was used as a marker for assessing the level of oxidative stress in several studies^46,47^. On the same manner, CAT and SOD act as scavengers for ROS neutralizing their damaging oxidative stress^24^.

The results of oxidative stress biomarkers indicated that acute administration of LA caused increase in MDA levels in all the studied tissues (brain, liver, and kidneys). This increase was only significant in brain tissues, besides to brain and liver tissues of both genera following the subacute LA administration. The significant increase in MDA levels in brain tissues could be assigned to the increase of lipid peroxidation after administration of LA. Raouf et al*.*^48^ and Haliwell^49^ concluded that the brain is more sensitive to oxidative damage than other tissues for its insufficient aerobic metabolism along with extra ROS production, eventually leading to more free-radicals reactions initiations. The overproduction of MDA was parallel to the reduction of GSH, this goes in line with all the previous studies regarding oxidative stress, attributed to the relation between increased formation of ROS and protective detoxification role of antioxidants^50^. In all LA treated groups, lower levels of MDA were detected in *Acomys* in most of the evaluated tissues compare to *Mus*, which indicate stronger oxidative stress resistance for *Acomys* than *Mus*. The ability of *Acomys* to resist the oxidative stress may correlate with the high basal GSH content measured in almost all studied tissues of untreated controls.

The high basal GSH, CAT and SOD may scavenge ROS and inhibit lipid peroxidation. The results also showed that the reduction in MDA level in *Acomys* tissues is accompanied with reduction in DNA damage. Scavenging of ROS and inhibition of lipid peroxidation may protect the cellular macromolecules, including DNA, from oxidative damage.

LA toxicity was clearly detected through histopathological examination of both species following both: acute and subacute treatments, but it was noticeable to be mostly less severe in *Acomys*, suggesting it to have higher tolerance and resistance to toxicity-induced histological changes. LA neurotoxicity in *Mus* appears to be in line with the extreme oxidative damage detected earlier. The neurodegenerative changes, the vacuolization, disrupted shapes of cells, and irregularity of nuclei, all concur with the Saleh and Meligy findings of LA effects on cortex in adult albino rats^51^. All together, these correlations and histological changes could be attributed to the oxidative damage caused to critical biomolecules as lipids, proteins, and DNA, by the LA^52^. The tissue damage severity in liver samples of *Acomys* observed during histopathological examination following the subacute LA treatment, as well, goes in line with the high TM, the high MDA level, and the low GSH level detected compared with those of *Mus* results. On the other hand, the severity observed histologically in the *Acomys* kidneys samples following the acute LA treatment goes in line with the TM and MDA. This could be supporting to our hypothesis that indicates a relation between the lower size of *Acomys* kidneys and the higher sensitivity to toxicants, *Acomys* kidneys have high potentials to resist damage even though their sizes are not helpful as they need. Imagine it as an imbalance between the tendency for resistance and the tendency for accommodation to damage in brief period of time. Whatever the case is, our histopathological findings agree with the findings of Raouf et al. regarding the LA effects^48^.

In this study we also detected several differences in the expression of some genes believed to be inflammation-related in the brain tissue. Two of which are pro-inflammatory cytokines/chemokines which play important roles in immunity protection and injury repairing^53^. *IL-1β* is one member of the cytokine family most linked to innate immune responses that include the major inflammatory mediators^53,54^. As expected, *IL-1β* was overexpressed in brains of both species in all treated groups. These results go in line with the increased levels of this gene during systemic inflammation in CNS detected in previous studies^55^. The extreme significant overexpression following both acute and subacute LA treatments, compared to the other genes evaluated, supports the evidence provided for the localization of *IL-1β* receptor in the brain that indicates for its key role as an inflammatory stressor neuroregulator of CNS^56^. However, this upregulation was less in *Acomys* than *Mus* indicated as 379% and 375% in *Acomys* vs. 408% and 1294% in *Mus* following acute and subacute LA treatments respectively, suggesting higher resistance to LA induced inflammation. The same difference between *Acomys* and *Mus* levels of *IL-1β* was detected with *Brant* et al.^6^. The same applies for the chemoattractant *CXCl-1* levels following the subacute LA treatment where it was only significantly overexpressed in *Mus*. In contrast, it was not significantly altered in either species following the acute LA treatment. In consistent with the previous illustrated data, the protein expression of proinflammatory cytokine TNF-α in the brain cortex was significantly elevated in *Acomys* compared to *Mus*. Kanhaiya et al*.*^57^ illustrated that LA increase the release of TNF-α in brain tissue.

Additionally, *Notch* *2* expression was evaluated since it was proved to have key role in regulating innate immune and inflammatory responses^58^. Following the acute LA treatment, *Notch* *2* was downregulated significantly in both species, but the reduction was greater in *Mus* than in *Acomys* (−74% vs −20% respectively). The same result was obtained in previous study when mice were exposed to acute UVB irradiation^5^. On the other hand, *Notch* *2* was upregulated in both species following the subacute LA treatment, and the greater expression was in *Acomys* (99%) compared to *Mus* (39%). This ambivalence in *Notch* *2* expression between acute and subacute treatments may in part be attributed to the conflicting functions of Notch signaling in various regulations, including inflammation, proliferation, regeneration, and cell fate^59,60^, as it is believed that the various ligand-receptor complexes of Notch signaling lead to different proteolytic events based on the stimuli that triggered it, different stimuli subjecting different ligands and receptors activate different signaling pathways^58,61^.

In summary, this study describes for the first time the potential capacity of the spiny mouse (*Acomys* *cahirinus*) to resist genotoxicity, oxidative stress and inflammation induced by acute and subacute lead acetate treatment. The results reflect a strong adaptive and protective response that *Acomys* *cahirinus* has developed to cellular or genetic stressors. Further studies are needed to clarify the correlation between the potential capacity of *Acomys* to resist DNA damage, oxidative stress and inflammation and its exceptional capabilities of healing and regeneration.

## Materials and methods

All experimental procedures were performed in accordance with relevant guidelines and regulations.

### Experimental animals

A total of 20 *Acomys* *cahirinus* and 20 *Mus* *musculus* (20–30 g) of either sex, bred and maintained in the institutional animal house were used for the experiment. Mice were housed in well-ventilated cages, under naturally controlled environment of temperature, humidity, and standard day/night light cycles. The animals were allowed standard food pellets and water ad libitum*.* All mice experiments were carried out following the national guidelines for the care and use of animals. The study was approved by the institutional animal care and use committee (IACUC) at Cairo University with approval number (CU/I/F/86/20). Additionally, all animal studies were performed in accordance with ARRIVE guidelines.

### Lead acetate (LA) doses and treatment schedule

**Figure 8:**
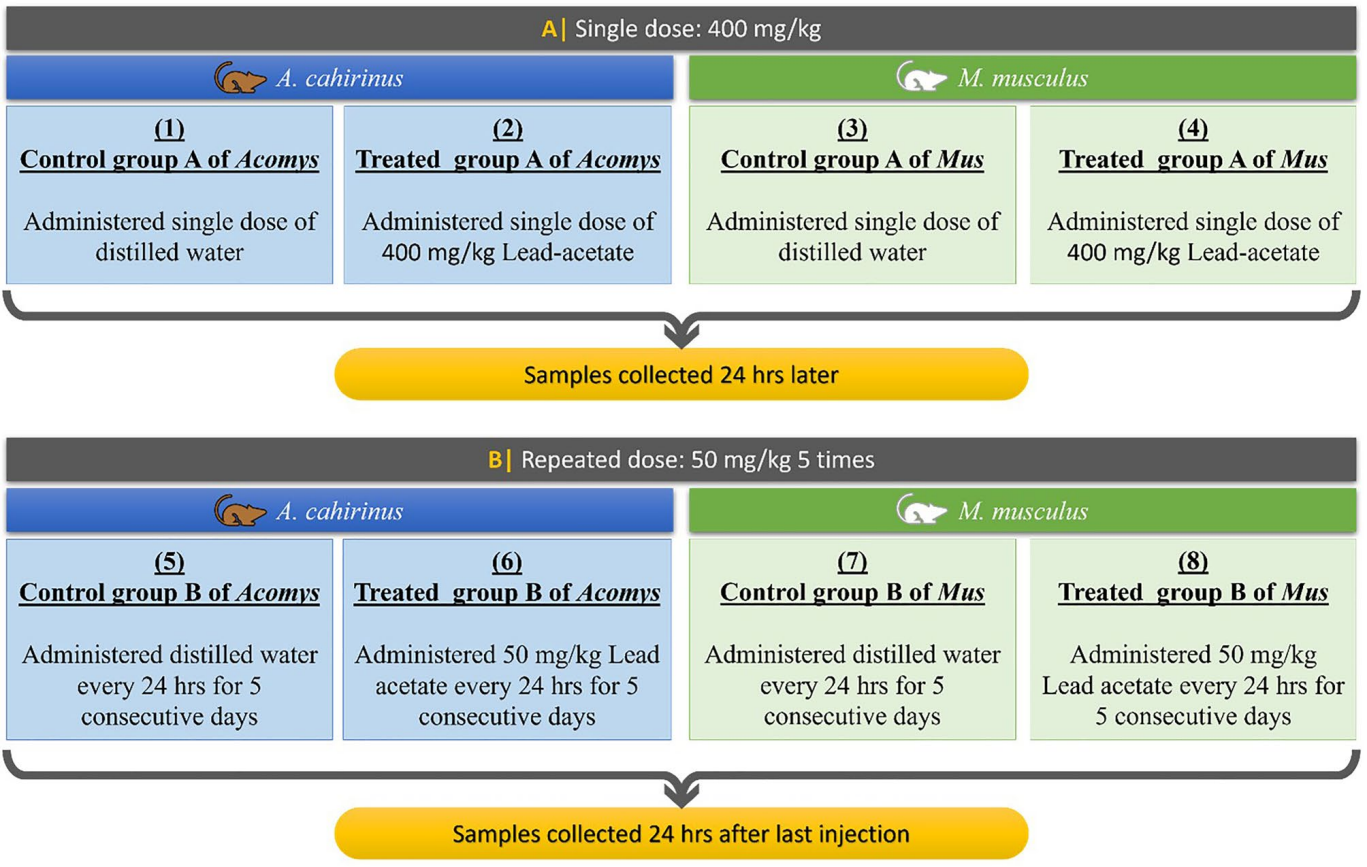
Experimental groups and the study design.

Lead acetate was purchased from Merck (Merck Company, Germany). LA was dissolved in distilled water and injected intraperitoneally either as a single dose (400 mg/kg) or as multiple doses (50 mg/kg for five consecutive days). The doses of LA were selected based on literature data^28,62^ and our preliminary experiments, as such doses are well known to exert a suitable biological response (induced genotoxicity, oxidative stress, and inflammation). Mice were sacrificed by cervical dislocation 24 h after the last LA administration. Details of treatment schedule are illustrated in Fig. 8.

### Sample collection

At the end of the experiment, the animals of each group were killed by cervical dislocation and their brain, liver, and kidney were carefully dissected, washed with cold PBS, then distributed as suitable-sized portions over prelabelled tubes for further analysis. Tissues stores in RNA later at -80ºC for gene expression analysis, at −20 °C for comet assay, and in buffered formalin for histopathological and immunohistochemical examination.

### Experimental outcomes

#### Comet assay

The extent of DNA damage was evaluated based on the protocol of alkaline single-cell gel electrophoresis (comet assay) described by Tice et al*.* 2000^36^. Slides were examined under fluorescent microscope (Leica, Germany). For each sample, the degree of DNA migration was determined by capturing images for 50 nucleoids at 400× magnification using leica microscope camera. Analysis of comet parameters was done by using TriTek Comet Score™ Version 1.5 software. Tail length (TL), %DNA in tail and tail moment (TM) are used to evaluate the extent of DNA damage.

#### Oxidative stress biomarkers

A 0.30–0.45 g weighted pieces of tissue samples collected were homogenized in 50 mM phosphate buffer (pH 7.4) and centrifuged for 10 min at 3000 rpm at 4 °C and the supernatants were then stored at −20 °C until used for determination of malondialdehyde (MDA), reduced glutathione (GSH) and antioxidant enzymes CAT and SOD in accordance to following protocols described by Ohkawa et al.^63^, Beutter et al.^64^, Aebi^65^, and Nishikimi et al.^66^*.*

#### Gene expression analysis

Brain samples that were conserved in RNA later at -80 ºC since eradication were used to assess the expression of the genes: *CXCL-1,* *IL-1β*, and *Notch* *2* using the *CDKn1-a* as a housekeeping gene for normalization. Primers were synthesized by Invitrogen (Carlsbad, CA, USA)^5^. RNA was extracted from the samples with RNeasy extraction kit according to manufacturer recommended protocol of (Qiagen GMbH, Germany). Purity and quantity of the RNA samples were assessed using Thermo Scientific NanoDrop 2000 to assure that concentrations were pure enough to conduct RT-PCR. cDNA was synthesized with an adjusted input of 1 µg of RNA extract using Reverse transcription kit (Thermo Fisher Scientific, RevertAid RT Reverse transcription Kit). RT-qPCR was performed using Master mix (SensiFAST ® SYBER No-ROX Kit) in RotorGene-6000 system (Corbett Robotics Australia) following the manufacturer protocol. For qPCR data analysis, Rotor-Gene Q series software (v.1.7) was used for a comparative quantification analysis. Relative gene expressions were calculated with the improved 2^-ΔΔCT^ method that directly uses the CT values obtained from the real time qPCR system^67^.

#### Estimation of histopathological parameters

Tissue samples collected were immediately fixed in 10% neutral buffered formalin for at least 72 h at room temperature. Later, following *culling* procedures and protocols for fixation and staining^68^, tissues from the brain cortex, liver, and kidneys were processes, sectioned into 5 μm thick sections using rotatory microtome, and stained with Hematoxylin and Eosin. After staining with hematoxylin and eosin, slides were examined under a microscope (Zeiss, Germany) at 100× magnification for histopathological changes.

#### Immunohistochemistry

Immunohistochemical examinations of inflammatory cytokine tumor necrotic factor alpha (TNF-α) was performed using streptavidin–biotin method. 5 microns thick paraffin embedded brain tissue section were prepared. Deparaffinized retrieved tissue sections were treated by 0.3% H2O2 for 20 Mins. Followed by brain samples were incubated anti TNF alpha (NBP1-19532) (1:200)-Novus Bio. Co. overnight at 4 °C. Tissue sections washed out by PBS followed by incubation with secondary antibody HRP Envision kit (DAKO) 20 min; washed out and incubated with diaminobenzidine (DAB) for 15 min. Washed by PBS then counter staining with hematoxylin, dehydrated and clearing in xylene then cover slipped for microscopic examination. According to adopted method form Heba et, al. 2022^69^. 6 non-overlapping fields were randomly selected and scanned from cerebral cortical regions of each sample by examiner histologist using Leica application module for histological analysis attached to full HD microscopic imaging system (Leica Microsystems GmbH, Germany); for the determination of relative mean area percentage of immunohistochemical expression levels of TNF alpha in cerebral cortical neurons.

#### Statistical analysis

The analyses of the present results were carried out using the Statistical package for social science (SPSS) software version 22. Data were displayed as (mean ± standard error). Independent t-test was applied to illustrate the statistical differences between the studied groups. *P* < 0.05 represent significant difference.

## Supplementary Information

Supplementary Figures.

## Data Availability

Raw data supporting the results reported in the manuscript are available with the corresponding author on reasonable request.
