# Supplementary material for: Comparative analysis of Acomys cahirinus and Mus musculus responses to genotoxicity, oxidative stress, and inflammation
Source: Sci Rep. 2023 Mar 9;13:3989. doi: 10.1038/s41598-023-31143-4 (PMC9998436; doi:10.1038/s41598-023-31143-4)
Supplement: Supplementary file 1 — Supplementary Figures. [file 41598_2023_31143_MOESM1_ESM.docx]

1. Alkaline comet assay gel pictures of liver tissues of *Acomys* and *Mus* treated intraperitoneally with (400 mg/kg and 50 mg/kg (5 consecutive days)) of lead acetate.


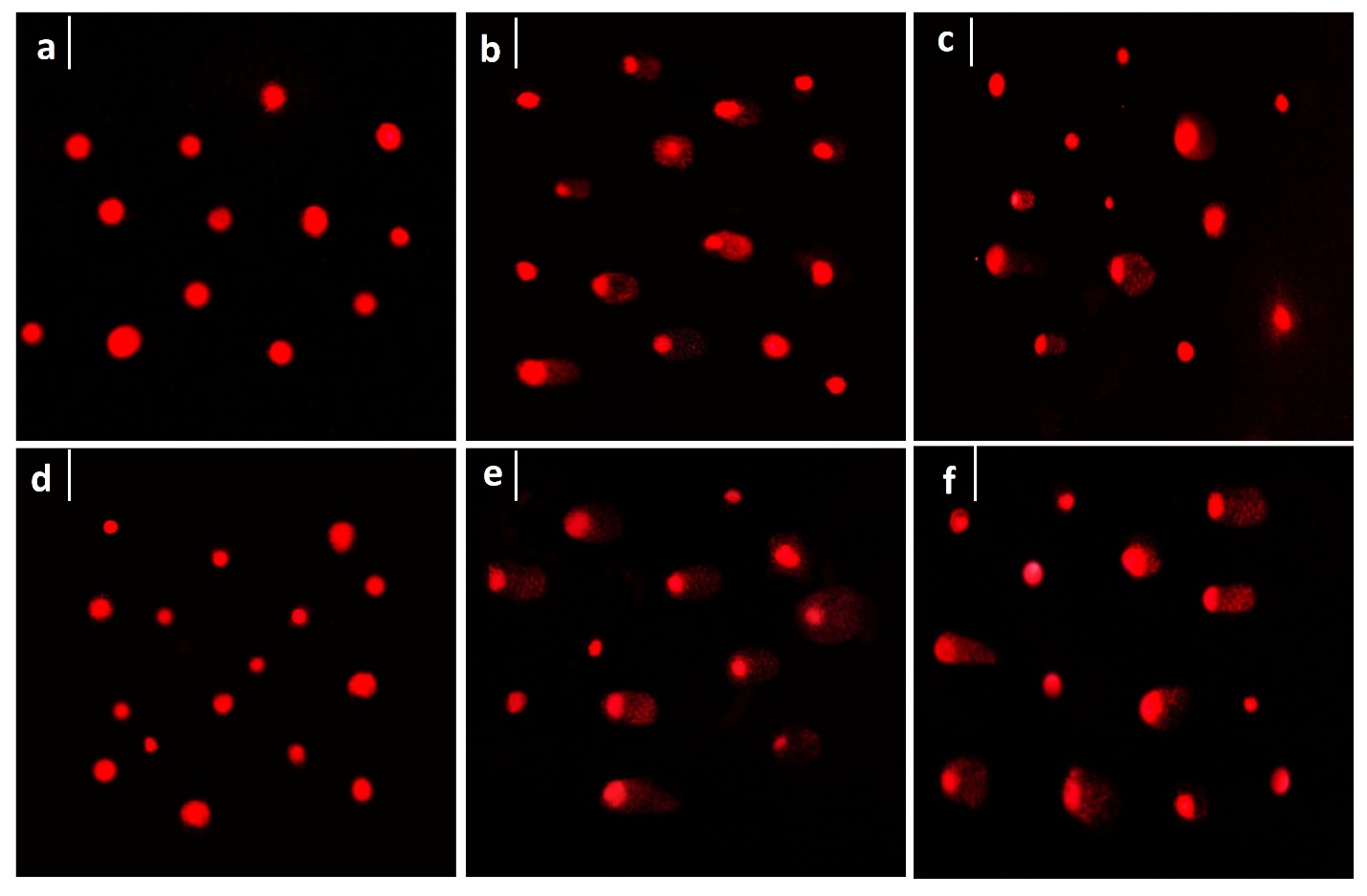
**Supplementary figure 1:** Fluorescent microscope photomicrographs represent the extent of DNA damage in liver using alkaline comet assay: (a) control *Acomys*, (b&c) treated *Acomys* with (400 mg/kg and 50 mg/kg) respectively, (d) control *Mus* and (e&f) treated *Mus* with (400 mg/kg and 50 mg/kg) respectively.

1. Alkaline comet assay gel pictures of kidney tissues of *Acomys* and *Mus* treated intraperitoneally with (400 mg/kg and 50 mg/kg (5 consecutive days)) of lead acetate.


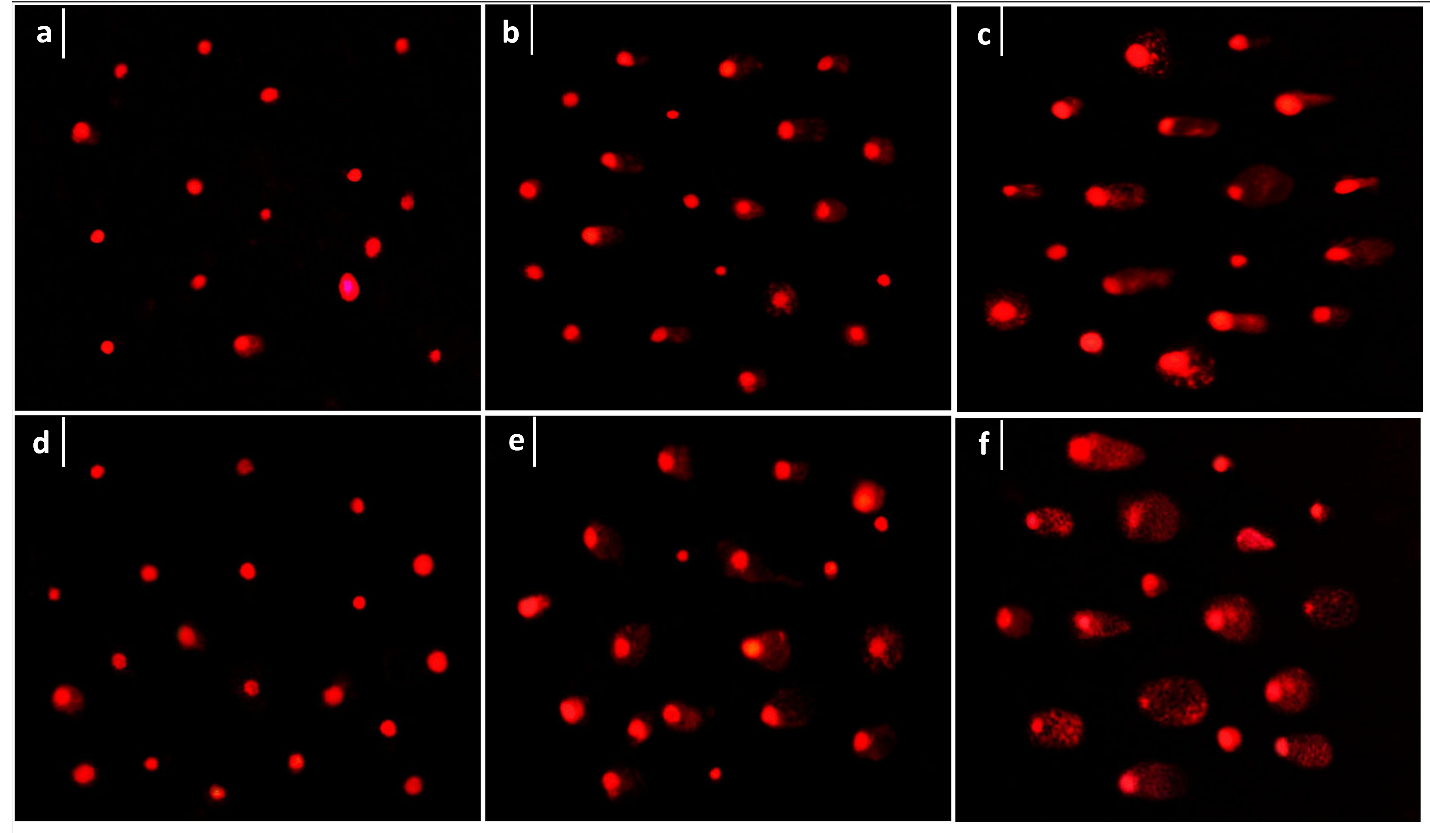
**Supplementary figure 2:** Fluorescent microscope photomicrographs represent the extent of DNA damage in kidney using alkaline comet assay: (a) control *Acomys*, (b&c) treated *Acomys* with (400 mg/kg and 50 mg/kg) respectively, (d) control *Mus* and (e&f) treated *Mus* with (400 mg/kg and 50 mg/kg) respectively.

1. Alkaline comet assay gel pictures of brain tissues of *Acomys* and *Mus* treated intraperitoneally with (400 mg/kg and 50 mg/kg (5 consecutive days)) of lead acetate.


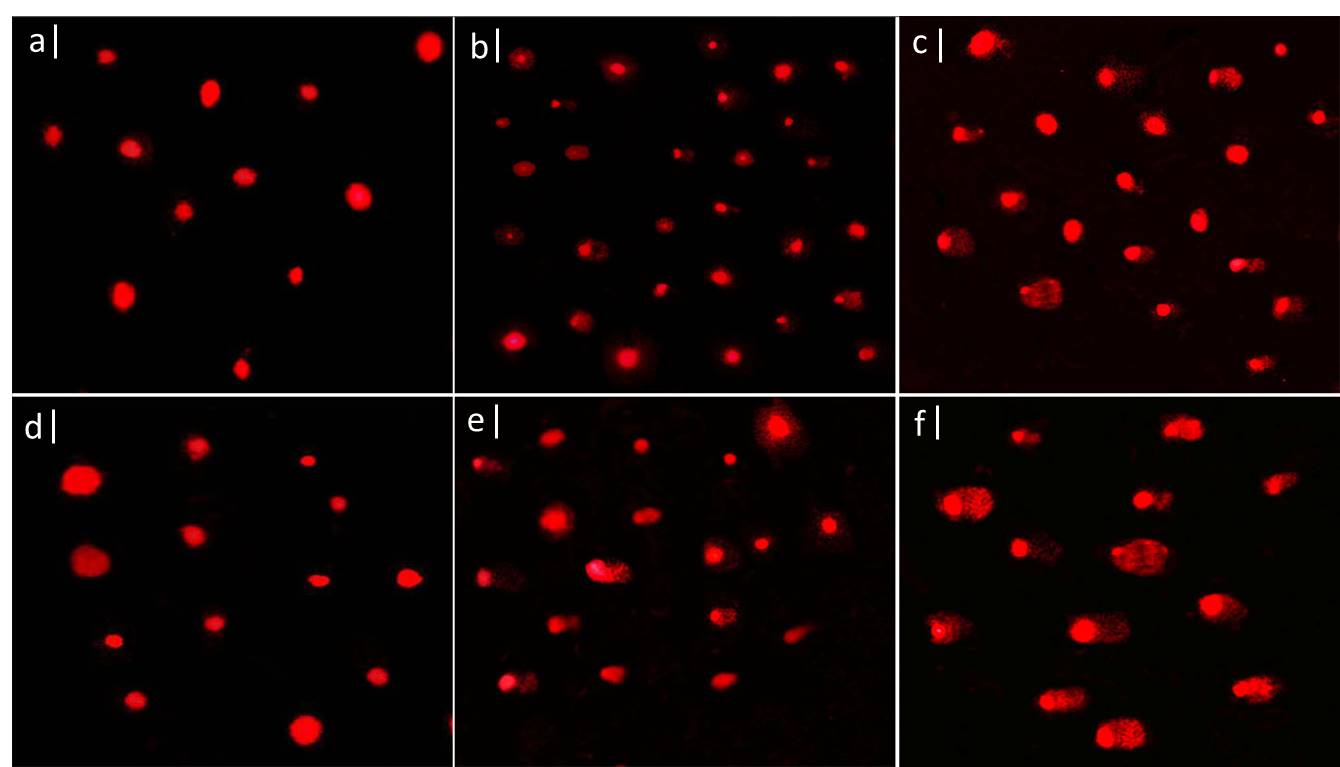
**Supplementary figure 3:** Fluorescent microscope photomicrographs represent the extent of DNA damage in brain using alkaline comet assay: (a) control *Acomys*, (b&c) treated *Acomys* with (400 mg/kg and 50 mg/kg) respectively, (d) control *Mus* and (e&f) treated *Mus* with (400 mg/kg and 50 mg/kg) respectively.
